# Changes in inpatient mental health treatment and related costs before and after flexible assertive community treatment: a naturalistic observational cohort study

**DOI:** 10.1186/s12888-025-06614-9

**Published:** 2025-02-25

**Authors:** Eva Brekke, Admassu N. Lamu, Renira C. Angeles, Hanne Clausen, Anne S. Landheim

**Affiliations:** 1 Research Centre for Substance Use Disorders and Mental Illness, Innlandet Hospital Trust, Brumunddal, Norway; 2 Department of Health and Social Sciences, Health Services and Health Economics Research Group, Norwegian Research Centre – NORCE AS, Bergen, Norway; 3 Department of Research and Development, Division of Mental Health Services, Akershus University Hospital, Lørenskog, Norway

**Keywords:** Flexible Assertive Community Treatment (FACT), Severe mental illness, Involuntary admissions, Hospitalization, Economic analysis

## Abstract

**Background:**

Flexible Assertive Community Treatment (FACT) is currently implemented in Norwegian mental health services, aiming to ensure comprehensive and rights-based services for persons with severe mental illness and complex needs, but also motivated by assumed cost-effectiveness. We need knowledge about the consequences of this service innovation. The aim of this study was to investigate changes in total and involuntary inpatient mental health treatment and associated changes in costs of inpatient days before and after enrolment into FACT for persons with severe mental illness and complex needs in Norway.

**Methods:**

In this naturalistic observational cohort study of 397 patients in eight Norwegian FACT teams, we compared total and involuntary admissions, total and involuntary inpatient days, and the costs of total and involuntary inpatient days, for two periods: 24 months before and 24 months after enrolment in FACT. We used paired t-test.

**Results:**

There was a significant reduction in involuntary admissions, involuntary inpatient days, and total inpatient days after enrolment in FACT. We found a slight but non-significant reduction in total admissions to inpatient mental health treatment. There was a significant reduction in the costs of total inpatient days and involuntary inpatient days.

**Conclusion:**

Patients in FACT were admitted to inpatient treatment as frequently as before enrolment in FACT, but involuntary admissions were less frequent. Furthermore, the duration of involuntary and total inpatient treatment was reduced, with a corresponding reduction in costs as expected. Results suggest that targeted and well-timed interventions from FACT may reduce the need for prolonged involuntary inpatient treatment, implying reduced disadvantages for the individual and more efficient allocation of health service funding.

**Supplementary Information:**

The online version contains supplementary material available at 10.1186/s12888-025-06614-9.

## Background

Despite attempts to reduce involuntary inpatient treatment, this is still widely used internationally [1] and in Norway [2]. Attempts to reduce involuntary treatment are justified through ethical and rights-based arguments, such as the right to health, autonomy, and genuine access to services [3, 4]. An additional argument for the reduction of inpatient treatment is the potential reduction in health service costs. While mental health services receive only a small percentage of total health budgets, mental health problems are among the costliest conditions in Norwegian health services. Costs of inpatient days, particularly the length of stay, constitute a large share of the total costs of specialist mental health services in Norway [5].

Norwegian health authorities have encouraged the implementation of models that allow for collaboration between different service levels, aiming to provide integrated care to a group of citizens who have otherwise received fragmented help with a high level of acute interventions. Health authorities have argued that assertive outpatient teams might ensure service user involvement and rights-based services, but also reduce unnecessary inpatient treatment and associated costs [6].

The Flexible assertive community treatment model (FACT) is a Dutch adaptation of Assertive community treatment (ACT) [7, 8]. These models aim to provide long-term, integrated and comprehensive services for persons with severe mental illness, complex needs, and a low level of daily functioning, who are not reached by traditional services. Supporting recovery and citizenship through assertive outreach are main treatment goals. Both FACT and ACT teams are multidisciplinary, consisting of psychiatrist, psychologist, nurses, social workers, peer support worker, job specialist and, in some teams, a music therapist. The teams work 80% of the time out of the office, with daily team meetings. The FACT model allows for a flexible variation between intensive and less intensive care, according to the patients’ needs, while ACT provides continuous intensive care. FACT has a broader target group than ACT, including persons who are not in need of continuous intensive care [9]. While severe mental illness is an inclusion criteria in both models, FACT inclusion criteria emphasize the level of functioning, including persons with a larger variety of diagnosis [10]. Inclusion is not based on former admission to inpatient treatment. The FACT model has a higher case load than ACT, but a smaller provider-patient ratio than regular services. The flexible variation between intensive and low-intensity care means that follow-up can continue at a low level during more stable periods, allowing for the prevention of crisis and swift intervention in the case of deterioration.

Implementation of ACT started in Norway in 2007, supported by health authorities [11]. While several ACT teams are still active, there has been a shift towards implementing FACT teams. Many Norwegian regions are scarcely populated, and FACT has been more applicable in rural regions due to the extended target group [12]. In addition, the FACT model is considered more cost-effective than the ACT model, although this has not been established by research [13]. Between 2013 and 2023, about 75 FACT teams were established in Norway. During the same period, there was a significant reduction in the mental health inpatient treatment capacity [14–16], partly justified with reference to the investment in FACT teams. To our knowledge, no previous research has investigated the association between enrolment in FACT and changes in inpatient treatment in a Norwegian context.

A naturalistic observational study of 142 patients in 12 different ACT teams in Norway found a reduction in inpatient days in the two years following inclusion in ACT, as compared to the two years prior to inclusion [11]. A reduction of total and involuntary inpatient days was found among patients with a high baseline use of inpatient services [17]. Patients with a low use of inpatient services at baseline had an initial increase in total inpatient days, leading the authors to hypothesize that ACT serves as a gateway to other services for some patients.

A Danish study found that persons who received services from FACT teams had fewer admissions and more outpatient contacts than persons who received services from community mental health teams (CMHT) or ACT teams [13]. However, there were no differences between groups in total inpatient days, use of coercion, episodes of self-harm, or deaths. Results from a qualitative study of service user experiences with coercion in FACT suggested that elements of the FACT model, such as long term, outreach, assertive, recovery-oriented and integrated follow-up, may prevent the need for coercion over time [18].

A study from the UK that compared the 12-month periods pre- and post-service change from Assertive Outreach (AO) into CMHT with FACT found that patients who received FACT had fewer admissions and inpatient days [19]. The authors concluded that FACT is a more cost-effective model than AO, based on a cost-sequence analysis. This conclusion was maintained in follow-up studies [20, 21]. An observational study in the UK found a reduction in inpatient days and fewer contacts with crisis resolution home treatment (CRHT) after CMHT treatment was replaced by FACT, when comparing a 13-month period six months prior to service change with a 13-month period six months after the service change [22]. Studies from the Netherlands have also found associations between inclusion in FACT and a decrease in admissions and inpatient days [9, 23].

There is a need for more knowledge about the association between FACT and involuntary and voluntary inpatient treatment in Norway. While FACT is considered to be associated with a reduction in involuntary and voluntary inpatient treatment, there is a lack of research that addresses whether there is an actual reduction in involuntary and total inpatient treatment for persons who are included in FACT in Norway. Economic arguments cannot be weighed against ethical and rights-based arguments. However, evidence concerning the efficacy of rights-based interventions may influence policy. Since a reduction in mental health service costs is among the health authorities’ arguments for implementing FACT and other models of integrated care, we need knowledge about the changes in costs of inpatient days after enrolment in FACT.

Hence, the aim of this study was to investigate the use of inpatient mental health treatment before and after enrolment into FACT, and associated changes in inpatient costs. Although there is a knowledge gap on the cost-effectiveness of FACT implementation in Norway and internationally, our study will not involve a full economic evaluation, but rather report a simple description of inpatient costs. The research questions were:

1. Are there changes in the use of inpatient mental health services (admissions, number of total/involuntary days) when comparing the situation 24 months before and 24 months after enrolment to a FACT team? The hypothesis is that enrolment in FACT will be associated with a reduction in total and involuntary admissions, and total and involuntary inpatient days.
2. Are there significant changes in the costs of inpatient days 24 months after the implementation of FACT? The hypothesis is that the cost of total inpatient days after enrolment in FACT is lower than the cost before enrolment to FACT. We hypothesize the same for involuntary inpatient days.

## Materials and methods

### Design

This was a naturalistic, observational cohort study designed as an uncontrolled before- and after study which was carried out in eight FACT teams in Norway. The study period ranged over ten years, from October 2011 to February 2021. Patients were included during 12 months after start-up of each team. The first team started in 2013, and the last team started in 2018. We compared data on an aggregated level based on individual means for the pre-enrolment period (24 months before enrolment) and the treatment period (24 months after enrolment). Since patients were included throughout the inclusion period, the data collection period in each team was two years before start-up and three years after start-up to cover 24 months treatment periods for all included patients. The timeline of data collection is depicted in Table 1.

**Table 1:** Timeline of data collection on inpatient mental health treatment pre- and post-enrolment for each team

Team	Data collection T0^a^		Data collection T1^a^		Inclusion period	
	Start date	Stop date	Start date	Stop date	Start date	Stop date
1	10/2011	09/2014	10/2013	09/2016	10/2013	09/2014
2	09/2012	08/2015	09/2014	08/2017	09/2014	08/2015
3	10/2012	09/2015	10/2014	09/2017	10/2014	09/2015
4	10/2012	09/2015	10/2014	09/2017	10/2014	09/2015
5	11/2013	10/2016	11/2015	10/2018	11/2015	10/2016
6	06/2014	05/2017	06/2016	05/2019	06/2016	06/2017
7	06/2014	05/2017	06/2016	05/2019	06/2016	05/2017
8	03/2016	02/2019	03/2018	02/2021	03/2018	02/2019

T0 = Pre-enrolment period (before enrolment in FACT); T1 = Treatment period (after enrolment in FACT)

^a^Since the inclusion period was one year, the data collection periods were three years to cover 24 months periods for all included patients

### Setting

The study was carried out in eight FACT teams in six hospital catchment areas in Norway. There are four health regions in Norway. Five teams in this study were in the Southwestern region, while three teams were in the Southeastern region. Five of the teams were in larger cities, one team was in a smaller city, while two were in rural areas. All teams were based on binding collaborations between specialized and municipal health services. The catchment areas varied from 20 000 to 120 000 inhabitants over the age of 18. All teams had been evaluated to have a high fidelity to the FACT model [8]. Structured evaluation had been carried out in each team by trained personnel from the Norwegian National Advisory Unit on Concurrent Substance Abuse and Mental Health Disorders, based on the FACT fidelity scale [24]. Total fidelity scores varied between 3.4 and 4.4. These scores are considered sufficient for certification as a FACT team according to the fidelity scale. Opening hours in the teams were between 0800 and 1530, leaving service users to attend acute services outside of opening hours, which is not in accordance with the FACT model.

Mental health treatment in Norway consists of two service levels, specialized and municipal services. Community mental health centres (CMHC) are part of specialized services and administered by the hospitals, while municipalities provide primary mental health services. Municipalities have a large degree of autonomy, and municipal services may vary. Both service levels share the responsibility for providing outpatient services to persons with severe mental illness and complex needs. Inpatient mental health treatment is managed by the hospitals, and may take place in acute wards, in hospital wards, or in CMHC wards.

The Norwegian Mental Health Act regulates the use of coercion in mental health services [25]. Coercion is only authorized when the person has a severe mental illness, voluntary treatment has been tried, and coerced treatment is considered necessary to prevent deterioration of the person’s health (the treatment criterion), or if the person may cause serious harm to others or to themselves (the danger criterion). Since 2017, if the person is considered to have the capacity to consent, coercion may only be authorized based on the danger criterion. Coercive treatment may be provided in an inpatient mental health facility (involuntary inpatient treatment) or in the community (community treatment orders [CTO]). Independent supervisory commissions are mandated to control the use of coercion in mental health services. There is geographical variation in involuntary psychiatric inpatient treatment in Norway, which has been explained by access to general practitioners, mental health nurses, and public housing in the communities [26].

### Eligibility

Teams 1–7 were selected because they were the first FACT teams in Norway. Team 8 was included later because it contributed to variation in population size and context, as this team was in a smaller city. All patients (400 persons) who had been enrolled in one of the eight FACT teams during the teams’ first year of operation, and who had remained with the team for at least 24 months, were included in the study. 29 persons had been admitted to one of the eight teams during the first year but remained in treatment for less than 24 months. These persons were not included in the study. The main reason for dropout was that the team could not establish contact with the person. Other reasons were relocation, death, that the person was no longer in need of FACT, or transfer to more intensive care. Three of the included 400 persons were in inpatient treatment during nearly the entire registration period. Data for these persons were removed from the material, based on the assumption that they had not received outreach services in the community from the FACT team. As a result, data for 397 persons have been included in the material.

### Data sources and measurement

Data on total admissions, involuntary admissions, total inpatient days, and involuntary inpatient days were registered for each patient for the pre-enrolment period (24 months before enrolment in FACT) and the treatment period (24 months after enrolment in FACT) (see Table 1).

Administrative staff in each FACT team collected the data from local electronic health records using a registration form developed by the research group. The data were anonymized and subsequently transferred to the research group. Admissions registered in the electronic health records as involuntary were counted as involuntary admissions, also those that were initiated as involuntary but changed to voluntary during the admission. Admissions registered as voluntary were counted as voluntary admissions. The sum of involuntary and voluntary admissions was counted as total admissions. This is in line with the registration practice in the Norwegian Patient Register.

The sum of involuntary and voluntary inpatient days was counted as total inpatient days. Inpatient days registered as voluntary were counted as voluntary inpatient days, while inpatient days registered as involuntary were counted as involuntary inpatient days. If the legal status (voluntary/involuntary) changed during an admission, this was registered to count the correct number of voluntary and involuntary inpatient days.

A healthcare resource use in terms of total costs of inpatient days and involuntary inpatient days was investigated, i.e., we report both the costs of total inpatient days and involuntary inpatient days. This will not give a full economic analysis of the FACT implementation but provide an evaluation of changes in the costs of inpatient days. For each FACT team, the costs per inpatient days were retrieved from the specialist health care reports by the Norwegian Directorate of Health [27, 28]. These include all costs related to the stay in the ward, including costs of staff. We used national average costs that may differ from FACT costs.

### Outcome variables

There are six outcome variables: *total admissions, involuntary admissions, total inpatient days, involuntary inpatient days, costs of total inpatient days,* and *costs of involuntary inpatient days*.

### Statistical analysis

Total and involuntary admissions, as well as total and involuntary inpatient days were presented as means for two time periods, the pre-enrolment period (24 months before enrolment in FACT) and the treatment period (24 months after enrolment in FACT).

Given different time intervals for the use of health services across the eight teams, costs of inpatient days were estimated at constant prices that adjust for price inflation (Appendix Tables A1 and A2). That is, costs were reported in 2021 prices using the consumer price index for health [29]. Eventually, costs of inpatient days are calculated as: *Number of inpatient days times costs per inpatient days*. The costs are reported in Norwegian kroner (NOK).

**Fig. 1:**
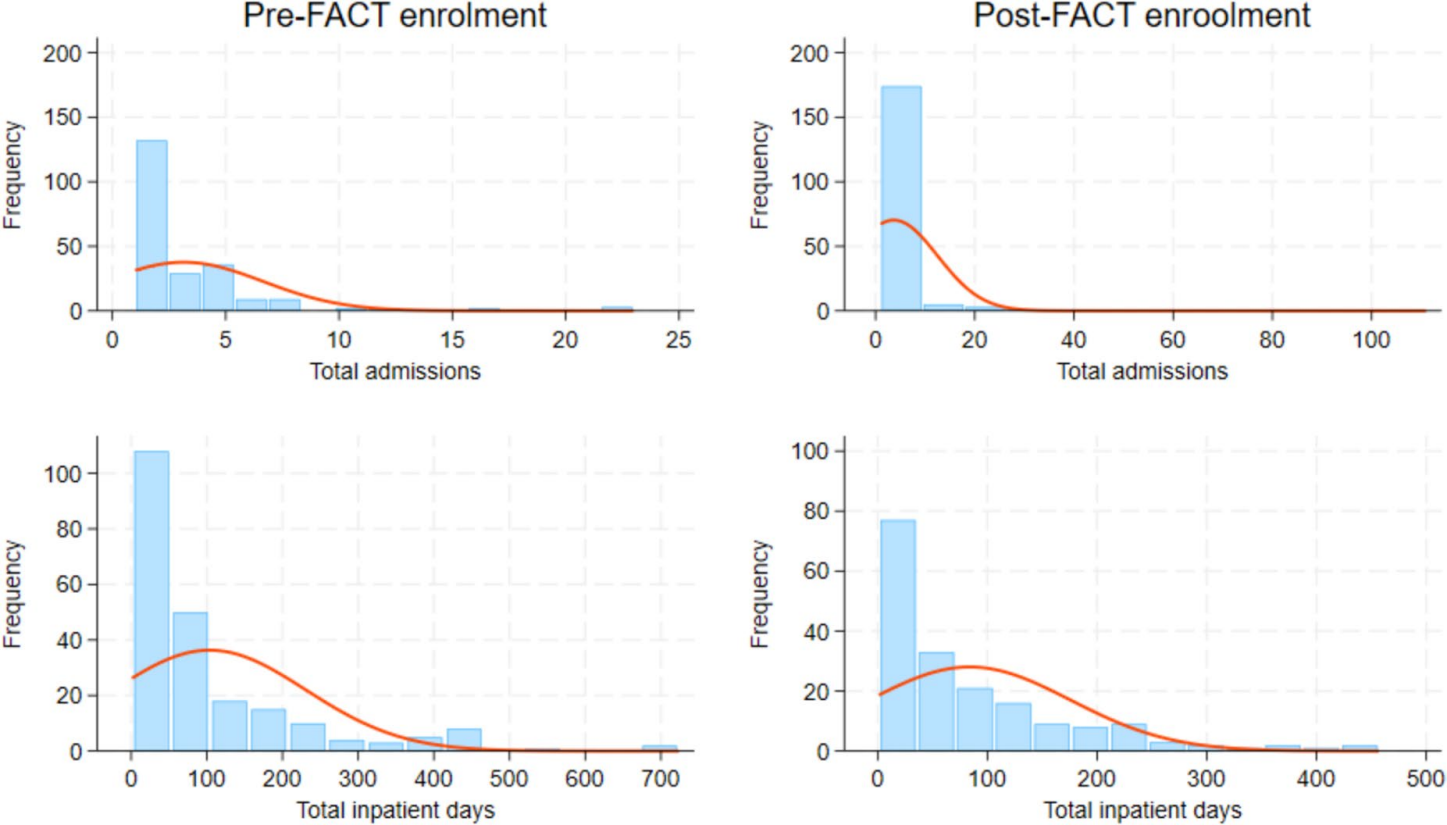
Distribution of total admissions and inpatient days in the pre- and post-FACT enrolment

**Fig. 2:**
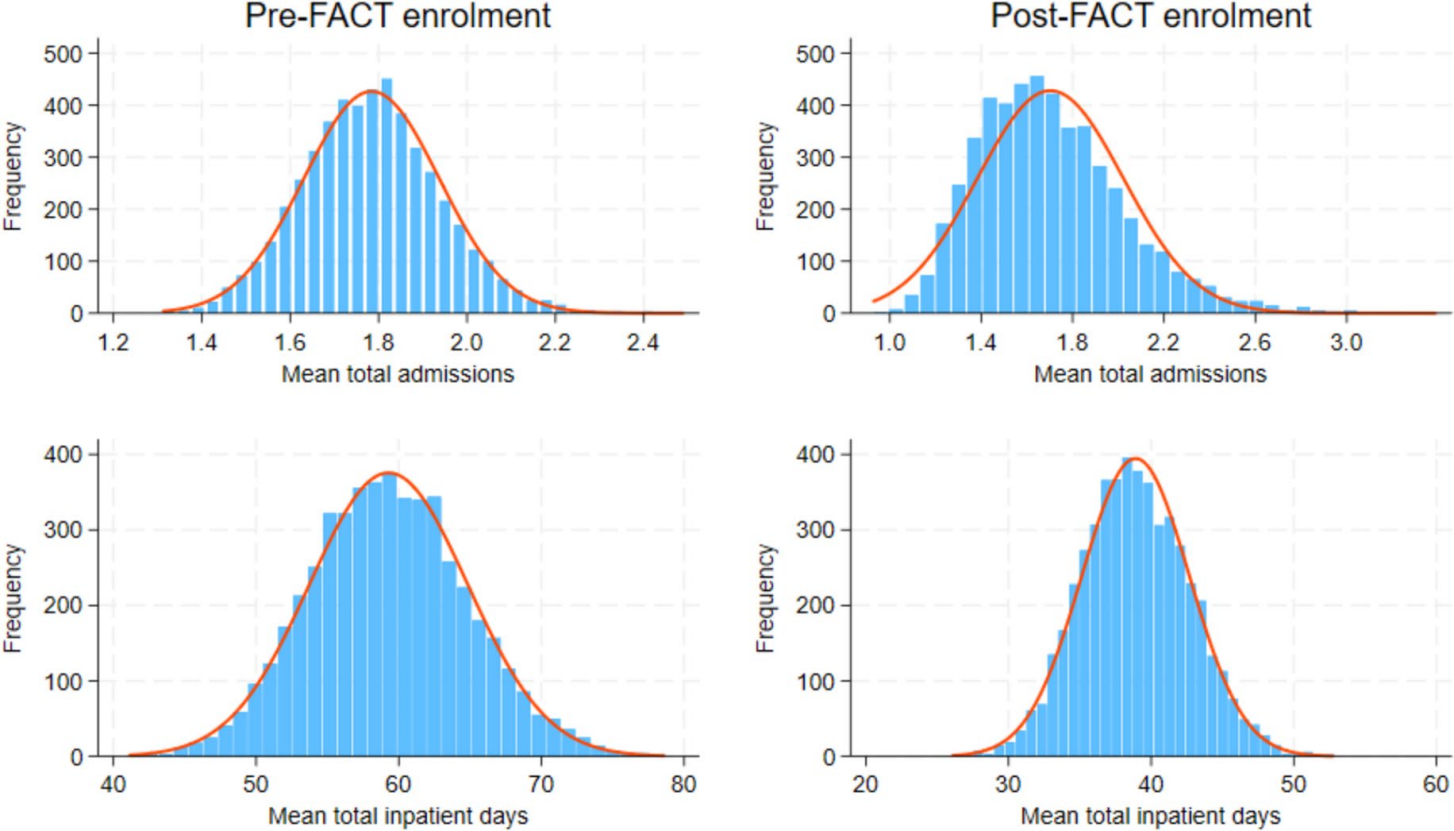
A non-parametric bootstrapped distribution of total admissions and inpatient days

Since the distributions of our data were more heavily skewed than normal, we used a non-parametric bootstrap test statistic to compare the means for total and involuntary admissions and total and involuntary inpatient days pre-post enrolment in FACT (See Figs. 1 and 2). We applied a more reliable bias and skewness adjusted (bias corrected and accelerated—BCa) method [30] with 5 000 bootstrap samples to calculate the confidence interval for differences in means of (total and involuntary) admissions and inpatient days. A similar test statistic was applied for total and involuntary inpatients costs. All analyses were conducted using Stata® ver. 18.0 (StataCorp LP, College Station, Texas, USA).

### Ethics

The study was approved by the local data protection officer (ID 137850). Since the study considers entirely anonymized data without any person-identifiable information, it was exempted from the requirement of informed consent. The research was conducted in accordance with the Helsinki Declaration of 1975, as revised in 2000 [31].

## Results

### Participants

Patient characteristics are presented on group level for each team (see Table 2). 128 persons (32%) had not been admitted to inpatient psychiatric care during the registration period. 83 persons (21%) had either 100 inpatient days or more, *or* five admissions or more, in the 24 months preceding enrolment in FACT.

**Table 2:** Description of sample by FACT teams

Team ID	1	2	3	4	5	6	7	8	Total
N (%)	80 (20)	35 (9)	81 (21)	93 (23)	33 (8)	24 (6)	24 (6)	27 (7)	397
Geography	Urban	Rural	Urban	Urban	Rural	Urban	Urban	Urban	-
Mean age	41	35	44	45	44	35	44	38	41
Women %	36	35	38	44	37	20	21	29	33
Diagnosis F20-F29%	90	60	94	87	59	65	54	34	68
Substance use problems	-	-	41	25	50	-	95	81	58
CTO %	-	-	47	35	44	-	44	4	35
High service users %	15	32	17	20	39	25	20	15	21

*-* = Missing information; *CTO* Community treatment order

### Admissions and inpatient days in the pre-enrolment period and the treatment period

Results for admissions and inpatient days (voluntary/involuntary) for the pre-enrolment period (24 months before enrolment in FACT) and the treatment period (24 months after enrolment in FACT) are presented in Table 3 and described below. The mean number of admissions and inpatient days for each team is presented in Appendix Table A3.

**Table 3:** Admissions and inpatient days pre- and post-enrolment in FACT (*N* = 397)

	Mean	S.E	BCa CI (95%)		*P*-values
			Lower	Upper	
**Total admissions**					
Pre enrolment period	1.78	0.15	1.51	2.13	
Treatment period	1.70	0.32	1.30	2.90	
Change (Pre -Post)	0.08	0.27	−0.82	0.46	0.769
**Involuntary admissions**					
Pre enrolment period	0.74	0.09	0.61	0.96	
Treatment period	0.43	0.05	0.35	0.53	
Change (Pre -Post)	0.31	0.09	0.17	0.51	0.000
**Total Patient days**					
Pre enrolment period	59.20	5.55	49.30	71.33	
Treatment period	38.89	3.77	32.32	47.07	
Change (Pre -Post)	20.30	6.01	9.34	32.79	0.001
**Involuntary patient days**					
Pre enrolment period	38.36	4.66	30.21	48.71	
Treatment period	21.76	2.92	16.69	28.13	
Change (Pre -Post)	16.60	5.25	6.31	26.89	0.002

BCa CI = 95% Bias corrected and accelerated bootstrapped confidence interval (with 5 000 repetitions)

S.E. = (Bootstrapped) standard error

During the pre-enrolment period, 224 (56%) persons had been admitted to inpatient psychiatric care, with a total number of 707 admissions. During the treatment period, 182 (46%) persons had been admitted to inpatient mental health treatment, with a total number of 675 admissions. There was a slight reduction in the total number of admissions after enrolment in FACT, but this change was not statistically significant. This may be due to one observation with a high number of admissions during the treatment period (111 admissions). If we omit this person, the mean number of admissions during the pre-enrolment period was 1.7 (95% CI: 1.44 to 2.01) and the mean number of admissions during the treatment period was 1.4 (95% CI: 1.12 to 1.72). The reduction in the total number of admissions was now approaching significance (*p* = 0.0515).

During the pre-enrolment period, 140 (35%) persons had been involuntarily admitted, with a total number of 295 admissions. During the treatment period, 103 (26%) had been involuntarily admitted, with a total number of 171 admissions. We found a statistically significant reduction in the number of involuntary admissions after enrolment in FACT (*p* < 0.001).

The total number of inpatient days during the pre-enrolment period was 23,232. The total number of inpatient days in the treatment period was 15,441. We saw a statistically significant reduction in the total number of inpatient days from the pre-enrolment period to the treatment period (*p* < 0.001).

The total number of involuntary inpatient days in the pre-enrolment period was 15,230. The total number of involuntary inpatient days in the treatment period was 8640. We saw a statistically significant reduction in the number of involuntary inpatient days from the pre-enrolment period to the treatment period (*p* < 0.002).

### Costs of inpatient days in the pre-enrolment period and the treatment period

Table 4 reports results of a non-parametric bootstrap test for the costs of total inpatient days and involuntary inpatient days in the pre-enrolment period and the treatment period. Panel-A gives estimates based on mean prices (of different teams). The reduction of costs of total inpatient days was significant (95% CI: 113 006 to 432 815 NOK) which confirmed our hypothesis that the costs of inpatient days were significantly lower two years after enrolment to FACT. The reduction in the cost of involuntary inpatient days was statistically significant (95% CI: 83 580 to 362 111). Results from Panel-B (estimates based on median prices) were quite like the findings from Panel-A.

**Table 4:** Costs of total and involuntary inpatient days pre- and post- enrolment in FACT (in NOK)

	Mean	S.E	BCa CI (95%)		*P*-values
			Lower	Upper	
***Panel-A: Estimates based on mean price***					
***Total inpatient days***					
Pre enrolment	799 346	75 022	665 779	963 477	
Treatment	536 670	51,996	446 226	650 002	
Change in costs	262 676	81 731	113 006	432 815	0.001
***Involuntary inpatient days***					
Pre enrolment	517 920	63 074	408 177	658 856	
Treatment	300 076	40 243	230 032	387 856	
Change in costs	217 845	71 425	83 580	362 111	0.002
***Panel-B: Estimates based on median price***					
***Total inpatient days***					
Pre enrolment	804 894	75 677	669 398	970 006	
Treatment	537 362	52 106	446 628	650,650	
Change in costs	267 533	82 307	117 918	439 262	0.001
***Involuntary inpatient days***					
Pre enrolment	521 639	63 576	410 927	664 125	
Treatment	300 532	40 334	230 424	388 561	
Change in costs	221 107	71 897	87 142	367 265	0.002

*BCa CI *95% Bias corrected and accelerated bootstrapped confidence interval (with 5 000 repititions), *S.E. *(Bootstrapped) standard error, *NOK *Norwegian kroner ($1 US ≅ 10.89)

## Discussion

The aim of this study was to investigate changes in voluntary and involuntary inpatient mental health treatment, and associated changes in costs, before and after enrolment into Norwegian FACT teams.

This study found no significant reduction in total admissions to inpatient mental health treatment after enrolment in FACT. However, there was a significant reduction in involuntary admissions, involuntary inpatient days, and total inpatient days after enrolment in FACT. Hence, the hypothesis that enrolment on FACT would be associated with a reduction in total and involuntary admissions, and total and involuntary inpatient days, was partly confirmed. This means that patients in FACT included in this study were admitted to inpatient treatment as frequently as before enrolment in FACT, but involuntary admissions were less frequent, and the duration of total and involuntary inpatient treatment was reduced.

Reduced frequency and duration of involuntary inpatient treatment after enrolment in FACT were significant findings from this study. These findings resonate with previous research that found associations between improved collaboration among specialist and municipal mental health services and reductions in involuntary inpatient treatment [26, 32]. In a qualitative study of patients’ experiences with coercion in FACT, participants described that safety and trust, improved quality of diagnosis and treatment, and increased participation and involvement had led to reduced involuntary treatment, and these experiences were facilitated by the focus on long-term, outreach, recovery oriented, multi-disciplinary and integrated services in the FACT model [18].

Early intervention in the case of mental health deterioration may prevent the need for coercive measures and long-term admissions, possibly through admissions of shorter duration at an earlier stage of a mental health crisis. While early admissions are increasingly difficult to access with less hospital beds available, it is possible that the FACT intervention increases the access to early admissions in some way. The above mentioned study described that close contact between patients and FACT personnel may lead to early detection of mental health deterioration and a reduced need for coercive measures [18]. A qualitative study of collaborating service providers’ experiences, including inpatient services, concluded that FACT teams may reassure other services and close existing gaps in the service system [33]. These are factors that might increase the access to early admissions for patients who are enrolled in FACT. This would be a possible explanation of the significant reduction in involuntary admissions, while the number of total admissions remained unchanged.

The inpatient treatment capacity in Norwegian mental health services has been reduced since 2013 [15]. Reduced inpatient capacity may indirectly lead to a reduction in inpatient treatment, but also an increased use of coercion if treatment is delayed or patients are discharged too early, leading to a deterioration of mental illness. Results from this study suggest that enrolment in FACT did not lead to a reduced need for, nor access to, inpatient treatment. According to the FACT model, the team is present during inpatient treatment and may intensify its intervention upon discharge. This may explain the reduction in involuntary and total inpatient days found in this study, without an increase in involuntary admissions. These findings suggest that enrolment in FACT is not associated with a reduced need for inpatient treatment, but perhaps a more adequate use of inpatient care that facilitates collaboration and voluntary treatment.

Findings from this study resonate with Norwegian studies that have investigated changes in inpatient treatment after inclusion in ACT [11, 17]. Results are not directly comparable due to different recruitment procedures. However, this similarity in results suggest that one might not expect large differences in how the two models affect changes in admissions, which is also in line with studies that have compared the two models [13]. Studies from ACT have shown positive outcomes in psychiatric symptoms, functioning and engagement, implying that a reduction in inpatient treatment is not associated with negative outcomes [34, 35]. There is a need for research that investigates such outcomes for patients in FACT.

Changes in legislation and efforts to reduce involuntary inpatient treatment may have impacted the results for the teams where the data collection period included the implementation of the changes in September 2017. Since the changes involved a confinement of the terms for authorizing the use of coercion, a reduction of involuntary admissions and inpatient days was expected. On a national level, there was a reduction in involuntary admissions between 2016 and 2017, but the rate of admissions per capita was back at the same level in 2018 as before the changes in legislation [36]. Registry data do not provide information about the total number of days in involuntary inpatient treatment, but reductions in CTOs and reduced activity of the supervisory commissions indicate a reduction in the duration of involuntary inpatient treatment since 2017. Since 2018, there has been an increase in the rate of involuntary admissions, but a reduction in the number of total admissions to inpatient mental health treatment in Norway [14, 37]. This trend is the opposite of results in this study. However, local trends in the catchment areas of each team may differ from the national trend. Hence, we cannot rule out that results are affected by changes in legislation, efforts to reduce coercion, or local trends in the use of inpatient treatment.

While data from some teams were missing, a substantial number of included patients had a CTO, which is in line with studies from ACT populations in other contexts [38]. While CTOs are considered by many a less invasive coercive measure than inpatient treatment, different stakeholders have mixed opinions of CTOs [39]. Qualitative studies have shown that CTOs may have substantial negative impact on the individual’s freedom and quality of life, also within a context of ACT and FACT, even if the ACT and FACT context may protect against some of the negative aspects of CTO [18, 40–42]. In one Australian study, CTO use was associated with reduced duration of inpatient treatment [43]. Meta-analyses have found no evidence that CTOs reduced the use of inpatient treatment [44, 45]. Others have warned against interpreting these findings as an argument against CTOs [46]. In a Norwegian study of regional differences in levels of involuntary treatment, lower levels of involuntary care, including CTOs, was not associated with a higher use of inpatient treatment [47]. While the benefits of CTOs remain a contested issue, results from the current study do not allow for conclusions about the relationship between CTOs and reductions in involuntary inpatient treatment, and CTO contribution cannot be ruled out.

This study found significant decreases in the costs of both total inpatient days and involuntary inpatient days. After enrolment to FACT, there was a significant reduction in total annual costs of inpatient days. The change in the costs of involuntary inpatient days had significant contribution to this change. This is not surprising, given the decrease in the number of inpatient days after enrolment in FACT. However, describing these changes in costs may serve to inform future policy for service development.

Enrolment in FACT may lead to better integrated, multidisciplinary community services that decreases the need for long- term inpatient care, which is in line with previous research [19]. Furthermore, closer follow-up as well as person-centred assistance by FACT team members after discharge would likely decrease the need for readmissions. Thus, the findings suggest that offering services from FACT teams may imply a sensible allocation of health resources in that efficient and well-timed interventions may prevent the need for prolonged intensive treatment. However, this study has only investigated costs of inpatient treatment, and one cannot rule out that there might have been increases in other health service costs.

### Strengths and limitations

Due to the descriptive nature of this study, we make no claims of causality nor prediction. The results should be interpreted in terms of association, and we cannot rule out the influence of other factors such as efforts to reduce coercion or local trends in inpatient treatment. Future research should include randomized controlled trials with pre-planned hypothesis and a control group.

A strength of this study is the inclusion of all patients in each of the eight teams, reducing the risk of selection bias. This differs from Norwegian studies of ACT, where inclusion was based on consent, which may imply selection bias [11]. However, collecting anonymized data means that the observed changes in admissions cannot be linked to other variables, which is a limitation of the current study. We did not collect data on previous treatment other than inpatient treatment. Hence, we do not know whether participants had previously been included in ACT, which would have been relevant information. To our knowledge, none of the catchment areas had ACT teams, but participants might have been enrolled in ACT teams in other regions.

Data were collected directly from patient records instead of a patient register. This, along with the use of a standardized spreadsheet for registration, and direct communication between the 5th author (AL) and team personnel who executed the registration, makes the data more reliable and rules out error due to inaccurate coding and reporting from hospitals.

A major weakness of the study is the descriptive nature of the data and the lack of a comparison group. Due to a lack of coding for FACT treatment in the National Patient Register, it has not been possible to generate a meaningful comparison group of persons in the same health regions, who did not receive services from FACT in the same period. This means that we cannot rule out the possibility that the observed reduction in involuntary admissions after enrolment in FACT is caused by other factors than the team intervention.

The small number of included teams compared to the total number of FACT teams in Norway may affect the generalizability of the results. Most teams were selected because they were the first teams in Norway, while only one team was selected based on considerations of variation. At the time of data collection, the teams were representative of the population, but we cannot generalize the results to all teams that were established later. Variation in admissions and inpatient days between teams may be interpreted in terms of sample characteristics, but also in terms of local variations in access to services and practice concerning inpatient (involuntary) treatment. Given the study design and small sample size in some teams, a comparison of results between teams is not considered appropriate and is hence not the aim of the current study.

Differences in changes of costs over time may not have been sufficiently detected. Complex derivation of costs, especially unit costs that involve different constant prices may produce bias and is prone to imprecision. Most importantly, we have not investigated all direct and indirect costs related to mental health care such as outpatient costs and out of pocket costs, which is required in a full cost-effectiveness analysis. As a result, we do not know whether other costs related to FACT may rule out the reduction in costs associated with inpatient treatment. Therefore, we make no claim to evaluate the economic benefits or disadvantages of the FACT model.

## Conclusion and implications

There was no significant change in the number of total admissions, but a significant reduction in involuntary admissions, involuntary inpatient days and total inpatient days after enrolment in FACT. The reduction in involuntary inpatient days was particularly large. As expected, we found a significant reduction in the costs of inpatient mental health treatment after enrolment in FACT, associated with the reduction in inpatient days. There is a need for future research with randomized controlled design to elaborate on the descriptive findings from the current study.

## Supplementary Information

Supplementary Material 1

Supplementary Material 2

## Data Availability

The datasets generated and analysed during the current study are not publicly available but are available from the corresponding author upon reasonable request.

## Abbreviations

FACT: Flexible Assertive Community Treatment
ACT: Assertive Community Treatment
CMHT: Community Mental Health Team
AO: Assertive Outreach
CRHT: Crisis Resolution Home Treatment
CMHC: Community Mental Health Centre
CTO: Community Treatment Order
