# Supplementary material for: Changes in inpatient mental health treatment and related costs before and after flexible assertive community treatment: a naturalistic observational cohort study
Source: BMC Psychiatry. 2025 Feb 25;25:164. doi: 10.1186/s12888-025-06614-9 (PMC11852869; doi:10.1186/s12888-025-06614-9)
Supplement: Supplementary file 1 — Supplementary Material 1 [file 12888_2025_6614_MOESM1_ESM.docx]

**Appendix Table A1.** Costs per inpatient days at constant prices

| Year | Costs/inpatient days**^Ϯ^** | 2021 constant price |
| --- | --- | --- |
| 2012 | 11455 | 12892 |
| 2013 | 11548 | 12997 |
| 2014 | 12132 | 13654 |
| 2015 | 12198 | 13729 |
| 2016 | 12295 | 13838 |
| 2017 | 12483 | 13779 |
| 2018 | 13843 | 13843 |
| 2019 | 13962 | 13962 |
| 2020 | 14820 | 14820 |
| 2021 | 14620 | 14620 |

**^Ϯ^** Costs per patient days from 2012 to 2017 are given at 2016 constant prices, and the rest at 2021 constant prices. These data were obtained from Health Directorate reports: *Produktivitet, aktivitet og ressursinnsats i psykisk helsevern og TSB* [Productivity, activity and resource use in mental health care and TSB]; and *Status og utviklingstrekk for spesialisthelsetjenesten* [Status and trends in the specialist health service] (avialbale at <https://www.helsedirektoratet.no/rapporter>). To convert costs in different years to constant 2021 prices, we used consumer price index (CPI) for health from Statistics Norway (SSB) - <https://www.ssb.no/en/statbank/table/03014/tableViewLayout1/>. For instance, the 2012-2016 costs (column-2) are converted to 2021 constant prices (column-3) as: (CPI2021/CPI2012)*Costs2012.

**Appendix Table A2.** Costs per patient per inpatient days by FACT teams at constant (2021) prices

|  | Pre-FACT enrolment | | Post-FACT enrolment | |
| --- | --- | --- | --- | --- |
| Teams | Median | Average | Median | Average |
| Team 1 | 12997 | 13181 | 13729 | 13740 |
| Team 2 | 13654 | 13460 | 13838 | 13782 |
| Team 3 | 13654 | 13460 | 13838 | 13782 |
| Team 4 | 13654 | 13460 | 13838 | 13782 |
| Team 5 | 13729 | 13740 | 13779 | 13820 |
| Team 6 | 13783 | 13740 | 13811 | 13820 |
| Team 7 | 13783 | 13740 | 13811 | 13820 |
| Team 8 | 13779 | 13820 | 13962 | 14208 |

For each team, data collection was conducted over several years (see Table 1). Here, we reported median and average costs of these periods using cost information from column-3 of Appendix Table-A1. For example, for Team 1, data collection took three years (≈ 2012-2014) in the pre-FACT enrolment. Hence, the median and average costs per inpatient days of these periods are 12 997 NOK and 13 181 NOK, respectively (which are obtained from the last column of Appendix Table A1). These median and average costs per inpatient days detailed in this Table A2 are used as unit costs (costs per patient per day) in the calculation of both total and involuntary costs of inpatient days reported in Table 4.
