# Supplementary material for: Changes in inpatient mental health treatment and related costs before and after flexible assertive community treatment: a naturalistic observational cohort study
Source: BMC Psychiatry. 2025 Feb 25;25:164. doi: 10.1186/s12888-025-06614-9 (PMC11852869; doi:10.1186/s12888-025-06614-9)
Supplement: Supplementary file 2 — Supplementary Material 2 [file 12888_2025_6614_MOESM2_ESM.docx]

**Appendix Table A3.** The mean admissions and inpatient days by the eight FACT teams

|  | Pre-enrolment | | | Post-enrolment | | | Cange |
| --- | --- | --- | --- | --- | --- | --- | --- |
|  |  | 95% CI | |  | 95% CI | |  |
|  | Mean (S.E.) | Lower | Upper | Mean (S.E.) | Lower | Upper |  |
| ***Involuntary admission*** |  |  |  |  |  |  |  |
| Team-1 | 1.00 (0.27) | 0.48 | 1.52 | 1.50 (0.35) | 0.81 | 2.19 | +0.50 |
| Team-2 | 1.08 (0.32) | 0.45 | 1.71 | 0.75 (0.15) | 0.46 | 1.04 | -0.33 |
| Team-3 | 1.48 (0.30) | 0.89 | 2.07 | 1.03 (0.19) | 0.65 | 1.41 | -0.45 |
| Team-4 | 1.00 (0.31) | 0.40 | 1.60 | 0.47 (0.23) | 0.01 | 0.92 | -0.53 |
| Team-5 | 1.22 (0.52) | 0.20 | 2.25 | 1.33 (0.45) | 0.46 | 2.21 | +0.11 |
| Team-6 | 2.56 (0.92) | 0.75 | 4.36 | 0.89 (0.35) | 0.20 | 1.57 | -1.67 |
| Team-7 | 1.44 (0.47) | 0.52 | 2.35 | 1.19 (0.45) | 0.30 | 2.07 | -0.25 |
| Team-8 | 0.67 (0.34) | 0.00 | 1.33 | 0.89 (0.27) | 0.36 | 1.42 | +0.22 |
| ***Total admission*** |  |  |  |  |  |  |  |
| Team-1 | 3.31 (0.70) | 1.94 | 4.68 | 3.65 (1.03) | 1.63 | 5.68 | +0.34 |
| Team-2 | 3.13 (0.56) | 2.02 | 4.23 | 2.46 (0.51) | 1.45 | 3.46 | -0.67 |
| Team-3 | 2.74 (0.46) | 1.84 | 3.64 | 2.81 (0.62) | 1.59 | 4.03 | +0.07 |
| Team-4 | 4.33 (1.41) | 1.58 | 7.09 | 10.67 (7.05) | -3.14 | 24.48 | +6.34 |
| Team-5 | 4.11 (0.64) | 2.85 | 5.37 | 5.22 (1.89) | 1.52 | 8.92 | -2.59 |
| Team-6 | 4.44 (0.99) | 2.50 | 6.39 | 3.11 (0.76) | 1.61 | 4.61 | -1.33 |
| Team-7 | 5.63 (1.50) | 2.68 | 8.57 | 4.38 (1.04) | 2.33 | 6.42 | -1.25 |
| Team-8 | 2.56 (0.55) | 1.48 | 3.63 | 5.78 (2.86) | 0.17 | 11.38 | +3.22 |
| ***Involuntary inpatient days*** |  |  |  |  |  |  |  |
| Team-1 | 46.38 (18.19) | 10.73 | 82.04 | 67.23 (16.79) | 34.33 | 100.14 | +20.85 |
| Team-2 | 40.54 (13.63) | 13.83 | 67.25 | 60.54 (18.97) | 23.35 | 97.73 | +20 |
| Team-3 | 100.97 (22.34) | 57.18 | 144.75 | 82.9 (18.38) | 46.89 | 118.92 | -18.07 |
| Team-4 | 59.13 (27.24) | 5.74 | 112.53 | 1.87 (1.19) | -0.47 | 4.21 | -57.26 |
| Team-5 | 43.44 (18.44) | 7.30 | 79.59 | 61.67 (29.81) | 3.25 | 120.08 | +18.23 |
| Team-6 | 84.56 (31.67) | 22.48 | 146.63 | 16.67 (7.88) | 1.23 | 32.10 | -67.89 |
| Team-7 | 47.25 (24.24) | -0.25 | 94.75 | 43.94 (16.27) | 12.05 | 75.82 | -3.31 |
| Team-8 | 47.22 (44.12) | -39.25 | 133.70 | 20.78 (13.37) | -5.42 | 46.97 | -26.44 |
| ***Total inpatient days*** |  |  |  |  |  |  |  |
| Team-1 | 81.15 (18.51) | 44.87 | 117.43 | 86.46 (16.34) | 54.44 | 118.48 | +5.31 |
| Team-2 | 72.04 (17.03) | 38.67 | 105.42 | 85.33 (21.1) | 43.98 | 126.69 | +13.29 |
| Team-3 | 131.71 (21.61) | 89.36 | 174.06 | 114.48 (19.21) | 76.83 | 152.14 | -17.23 |
| Team-4 | 142.6 (39.98) | 64.24 | 220.96 | 113.53 (32.65) | 49.55 | 177.52 | -29.07 |
| Team-5 | 139.33 (46.4) | 48.38 | 230.28 | 135.89 (34.5) | 68.27 | 203.51 | -3.44 |
| Team-6 | 109.11 (30.53) | 49.27 | 168.95 | 80.89 (18.83) | 43.98 | 117.80 | -28.22 |
| Team-7 | 97.69 (28.42) | 41.99 | 153.38 | 75.5 (18.23) | 39.77 | 111.23 | -22.19 |
| Team-8 | 81.89 (44.47) | -5.26 | 169.04 | 57.89 (22.51) | 13.77 | 102.01 | -24.00 |

Mean involuntary admission for Team-6 is significantly different from each of the other teams in pre-FACT enrolment, but no significant difference observed in the post-FACT enrolment. For total admissions, Teams 1, 2, 3, and 8 showed significant differences from Team-6 and Team-8 in the pre-FACT enrolment; and Team-4 has significantly higher mean compared to teams 1, 2 & 3. Significant mean difference is observed between Team-1 & Team-3 for involuntary inpatient days in the pre-FACT enrolment, & significant difference between Team-1 & Team-4, as well as Team-3 and teams, 4, 6, and 8 in the post-FACT enrolment. For total inpatient days, no significant difference observed in post-enrolment period, but Team-4 showed higher mean values compared to each of the remaining teams.
